# Social inheritance of avoidances shapes the structure of animal social networks

**DOI:** 10.1093/beheco/arad088

**Published:** 2023-10-23

**Authors:** Celine H Frère, Barbara Class, Dominique A Potvin, Amiyaal Ilany

**Affiliations:** School of the Environment, University of Queensland, St. Lucia, QLD 4072, Australia; School of Science, Technology and Engineering, University of the Sunshine Coast, Petrie, QLD 4502, Australia; Department of Biology, Ludwig-Maximilians-Universität München, Großhaderner Straße 2, 82152 Planegg-Martinsried, Munich, Germany; School of Science, Technology and Engineering, University of the Sunshine Coast, Petrie, QLD 4502, Australia; Faculty of Life Sciences, Bar-Ilan University, Ramat Gan 590002, Israel

**Keywords:** animal social networks, cultural evolution, social inheritance, social structure

## Abstract

Social structure can have significant effects on selection, affecting both individual fitness traits and population-level processes. As such, research into its dynamics and evolution has spiked in the last decade, where theoretical and computational advances in social network analysis have increased our understanding of its ecological and inheritance underpinnings. Yet, the processes that shape the formation of structure within social networks are poorly understood and the role of social avoidances unknown. Social avoidances are an alternate of social affiliation in animal societies, which, although invisible, likely play a role in shaping animal social networks. Assuming social avoidances evolve under similar constraints as affiliative behavior, we extended a previous model of social inheritance of affiliations to investigate the impact of social inheritance of avoidances on social network structure. We modeled avoidances as relationships that individuals can copy from their mothers or from their mother’s social environment and varied the degrees to which individuals inherit social affiliates and avoidances to test their combined influence on social network structure. We found that inheriting avoidances via maternal social environments made social networks less dense and more modular, thereby demonstrating how social avoidance can shape the evolution of animal social networks.

## INTRODUCTION

Social structure, the backbone of sociality, can have significant effects on selection (Whitehead et al. 2019). An individual’s place in the social scaffolding of its population can affect its fitness, including survival (Silk et al. 2003, 2009; Stanton and Mann 2012) and reproductive success (Frère et al. 2010). On a broader scale, social structure can also influence population dynamics, playing a role in genetic evolutionary processes (Whitehead 1998, 2017), information transmission (Duboscq et al. 2016; Firth et al. 2016) and disease spread (Sah et al. 2017, 2018a,b). As such, research into the dynamics and evolution of social structure has spiked in the last decade, where theoretical and computational advances in social network analysis have advanced our understanding of its ecological and inheritance underpinnings (Ilany and Akçay 2016; Ilany et al. 2021).

Classic definitions of sociality refer to both the social attraction to, and avoidance of, conspecifics. This suggests that like affiliative behavior, social avoidance is integral to the evolution of sociality and likely plays a key role in shaping social structure (Bejder et al. 1998; Whitehead 1999, 2009a; Whitehead et al. 2005; Aplin et al. 2013; Whitehead and James 2015). And while it is generally accepted as an alternate to social affiliation in animal societies (Whitehead et al. 2005; Whitehead 2009b), our knowledge about the evolution of social avoidance is limited to short-term or highly contextualized withdrawals, such as inbreeding avoidance (Archie et al. 2007) sex segregation (Wielgus and Bunnell 1994; Galezo et al. 2017), avoidance of diseased conspecifics (Loehle 1995; Behringer et al. 2006), dominance hierarchies (Carter et al. 2016), and social foraging interactions (Harten et al. 2018). Importantly, these studies highlight that avoidance is an active social behavior, separate from random temporal or spatial patterns that might simply signal a lack of preference or indifference to other individuals.

Astoundingly, however, we still know very little about long-term social avoidances (hereafter social avoidances), whereby animals actively and consistently avoid certain conspecifics throughout most, if not all, of their lifespan (Strickland et al. 2017). There are likely two reasons for this. The first is that avoidances may be incorrectly interpreted as simply “lack” of social preferences (designating them a passive rather than active behavior), despite contradicting evidence. Second, unlike social affiliations and short-term withdrawals, long-term social avoidances are not easily observed, and must be parsed from a lack of social interaction despite shared use of space (Strickland et al. 2017). In this sense, they hide in plain sight, forming an invisible web of aversive or circumventive non-connections, which like affiliative behavior, have the potential to play a central role in structuring social networks and population dynamics.

While social avoidances have up until recently remained in the shadows, they nonetheless are a phenomenon that occurs across the animal kingdom (Strickland et al. 2017). A basic search for studies, including those utilizing the Socprog program (Whitehead 2009b) revealed 31 publications across 30 vertebrate taxa specifically identifying social avoidances among conspecifics, even just from this limited subsampling of the literature (Supplementary Table 1). Indications of the presence of social avoidances have been found, for example, in primates (Henzi et al. 2009; Smith-Aguilar et al. 2016; Surbeck et al. 2017), New Caledonian crows *Corvus moneduloides* (Rutz et al. 2012), giraffes *Giraffa camelopardalis* (Carter et al. 2013); sperm whales *Physeter macrocephalus* (Gero et al. 2008); and sleepy lizards *Tiliqua rugosa* (Leu et al. 2010). Additionally, recent research has confirmed that social avoidances are indeed an active social decision similar to preferred affiliations (Strickland et al. 2017). Last and most importantly, we now know that individuals can modify their number of social avoidances as a response to the extent of competition/conflict they experience (e.g., higher density living and aggression (Piza-Roca et al. 2018; Strickland et al. 2018); indicating that social avoidance likely evolves under the same constraints as affiliative behavior. As such, we argue that unraveling the evolutionary information avoidance patterns contain will help us better understand how social structures emerge and are maintained in nature.

Here, we use simulations to test the hypothesis that long-term social avoidances are key to the formation of structure in social networks. Assuming that socially avoiding certain individuals is as important as affiliating with others, social avoidances should behave like affiliation and be socially inherited (Fowler et al. 2009; Ilany and Akçay 2016), which was modeled via two different processes. The first one was directly adapted from the maternal inheritance model of Ilany and Akçay (2016), in which newborns copied their mother’s relationships (affiliates and avoidances) with a certain degree of fidelity (one-step model). In the second, “two-step” model, newborns inherited social networks from both their mother and her social environment. While maternal links are often important in the animal kingdom and can determine an individual’s social network early in life, individuals may also learn who to associate with or to avoid from other individuals present in their mother’s social environment. Similarly, an individual immigrating to a new population may copy associations or avoidances from one or several non-independent “role models.” We then tested how various degrees of social inheritance of associates and avoidances impact different measures of network structure.

## METHODS

### Model description

Our models were adapted from previous generative models (Jackson and Rogers 2007; Ilany and Akçay 2016) in which “role model” individuals introduce their social network to newborn individuals. In these simulations, these role models and new individuals were assumed to be mothers and their offspring, respectively. In order to maintain a stable population size *N*, both birth and death occurred at equal rates: in each time step, one randomly sampled individual died and another randomly sampled individual produced one offspring. We modeled undirected networks in which pairs of individuals could either associate (1), avoid each other (−1), or not have any relationship (0). Mothers and their offspring were assumed to always associate.

The inheritance of social relationships was modeled in two ways: in the first (“one-step”) model, offspring individuals only inherited their mother’s social network while in the second (“two-step”) model, offspring individuals first inherited their mother’s social network and then inherited their new associates’ social networks. Therefore, the inheritance of social networks was only vertical in the first model while in second model, it included horizontal inheritance. In both models, the fidelity with which networks were inherited was determined by the parameters Pn and Pa, for associates and avoidances, respectively. When associates and avoidances failed to be inherited (1-Pn and 1-Pa, respectively) relationships between these individuals and the offspring were null (0). Additionally, offspring were allowed to form new relationships with individuals that are not associates or avoidances of their role models, which could be positive or negative, both with a probability determined by Pr (1–2*Pr otherwise).

**Figure 1:**
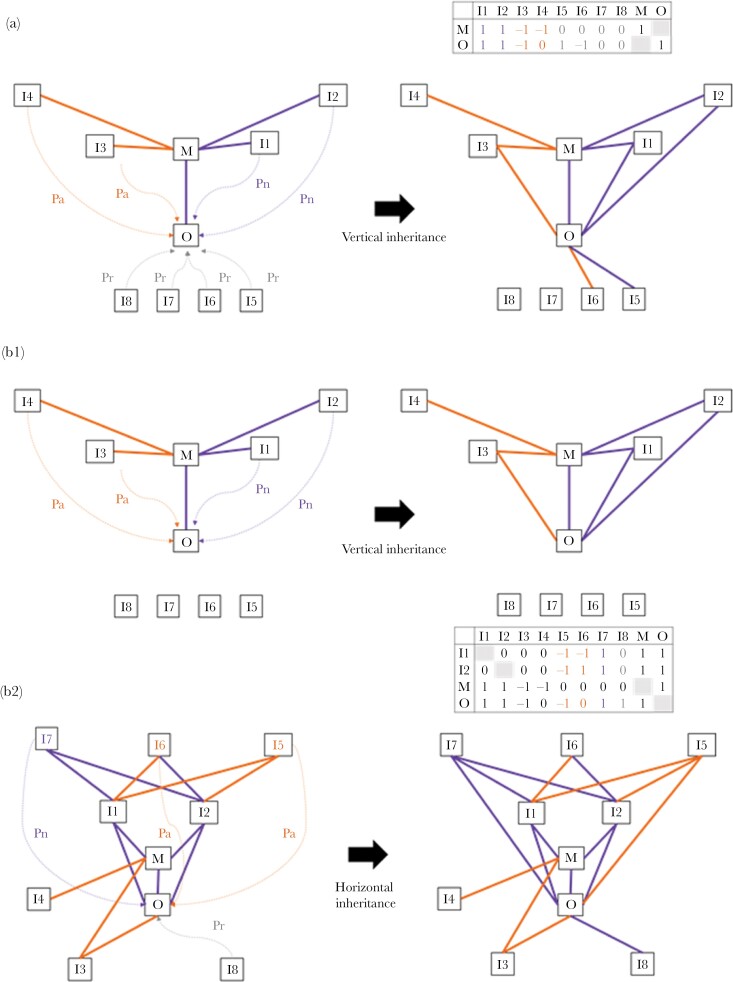
Graphical illustration of the models. In the first model (Figure 1A), each offspring (O) inherits its mother’s (M) associates (purple links) and avoidances (orange links) with probabilities Pn and Pa, respectively (dashed arrows) and forms new relationships with probability Pr. In this example, the offspring inherited all its mother’s associates (I1 and I2), only half of its mother’s avoidances (I3 but not I4) and made a new associate (I5) and a new avoidance (I6). The resulting network was also presented as a matrix of relationship between O, M, and the other individuals in the network. Avoidances and associates are presented as -1 and 1, respectively, while null relationships are zero. In the second model, the offspring first inherits its mother’s network (Figure 1 B.1) before inheriting its associates’ network and forming new relationships (Figure 1 B.2). Potential associates had more positive than negative relationships with the offspring’s associates (I7 in this example) and were inherited with a probability Pn. Potential avoidances had a number of negative relationships with the offspring’s associates at least equal to the number of positive relationships (I6 and I5 in this example) and were inherited with a probability Pa. Individuals that had no relationship with any of the offspring’s associates could form any type of relationship with the offspring with probabilities determined by Pr. In this example, the offspring first inherited all its mother’s associates and avoidances as in A, and then inherited its associates’ only associate (I7), half of their avoidances (I5 but not I6) and formed a new associate relationship with I8. The resulting network was also presented as a matrix of relationship between O, M, their associates I1 and I2, and the other individuals in the network.

In the first model, an offspring hence inherited its mother’s associates (1) and avoidances (−1) with probabilities Pn and Pa, respectively and could randomly form new relationships of any type with individuals that were not directly connected to its mother, with probabilities determined by Pr (Figure 1A).

In the second model, an offspring first inherited its mother’s associates and avoidances with probabilities Pn and Pa, respectively (as in the first model, Figure 1B.1). In the second step, the offspring formed ties with the associates and avoidances of its maternally inherited associates (Figure 1B.2). To allow this horizontal social network inheritance, each potential new social partner was first classified as either a potential associate, as potential avoidance, or a “neutral” individual. A potential associate was an individual that associated positively with more than 50% of the offspring’s associates. This potential associate became the offspring’s associate with a probability Pn, or alternatively, did not form any tie to the offspring with a probability 1-Pn. A potential avoidance was an individual avoided by at least 50% of the offspring’s associates. This potential avoidance became the offspring’s avoidance with a probability Pa, or alternatively, did not form any tie to the offspring with a probability 1-Pa. A “neutral” individual was an individual that had no relationship with any of the offspring’s associates (or mother) and could thus form any type of relationship with the offspring. This individual could thus become an associate with a probability Pr, an avoidance with a probability Pr, or have no relationship with the offspring, with a probability 1–2*Pr.

Simulations were run for a population consisting of 50 individuals. At time zero, a symmetrical relationship matrix of dimensions N × N was randomly generated, with probabilities of association, avoidance, and non-relationship of 0.1, 0.1, and 0.8, respectively. As in Ilany and Akcay (2016), we set the number of timesteps to 1000 to allow the networks to stabilize, which was also verified visually. These simulations were replicated 100 times for 162 different sets of parameters. Parameter sets were defined to test the impacts of socially inheriting avoidances with varying fidelity values (Pa varied from 0, i.e., absence of inheritance, to 0.8 with increments of 0.1). These impacts were furthermore tested in different contexts as we varied the intensities of Pn (from 0.3 to 0.8, with increments of 0.1) and Pr (which was either 0.001, 0.01, or 0.05), to mimic the range of values found in empirical systems by Ilany and Akcay (2016).

For each replicate and at each time step, we estimated the number of associate and avoidance relationships from the generated network and extracted metrics to describe the structure of the network of associations (removing all avoidances). First, the global clustering coefficient was calculated as the network’s global transitivity in its weak form (i.e., the probability that A and C form a tie given that A and B and B and C are linked) using the function gtrans from the R package sna (Butts 2020). Higher values of the clustering coefficient mean that nodes tend to cluster together more. Modularity describes the extent to which the network is partitioned into discrete communities. These communities were detected using a cluster walktrap community-detection algorithm from the R package igraph (Csardi and Nepusz 2006). Higher values of modularity mean that there are denser connections within than between communities (Newman 2006; Shizuka and Farine 2016).

## RESULTS

### Vertical transmission of social relationships (one-step model)

**Figure 2:**
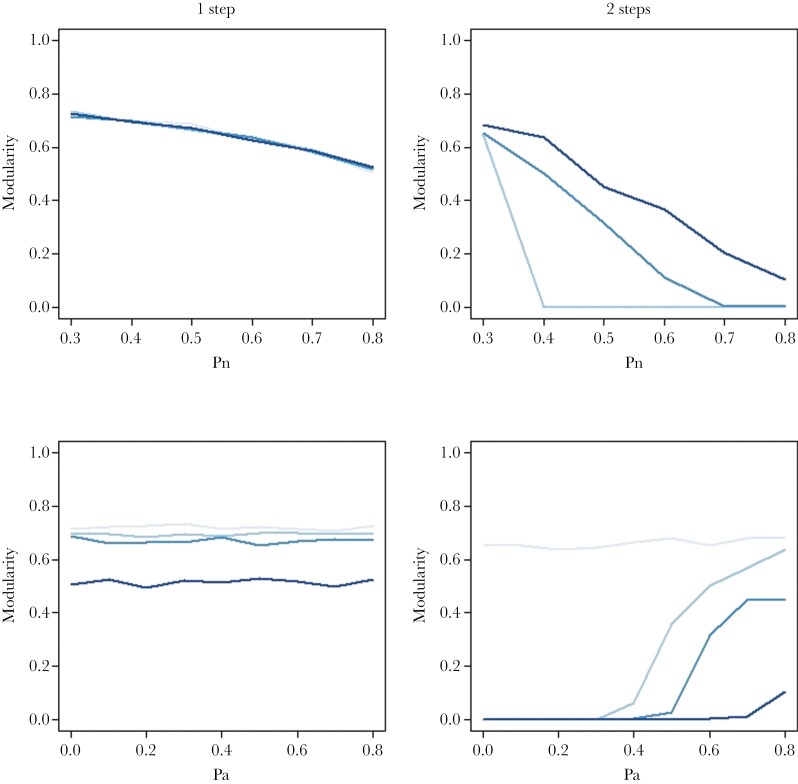
Modularity as a function of two social inheritance models and their parameters. Modularity was estimated at the last time step of each simulation and averaged across 100 replicates for the one-step model (or “vertical inheritance,” left panels) and the two-step model (or “vertical + horizontal inheritance,” right panels). For the two top panels, the four curves (from light to dark blue) correspond to different intensities for the social inheritance of avoidances (Pa = 0, 0.3, 0.6, 0.8). For the two bottom panels, the four curves (from light to dark blue) correspond to different intensities for the social inheritance of associates (Pn = 0.3, 0.4, 0.5, 0.8). For all panels, the probability to form random new ties (Pr) was 0.01, network size was fixed to 50 individuals, simulations were initiated with random networks and run for 1000 time steps.

**Figure 3:**
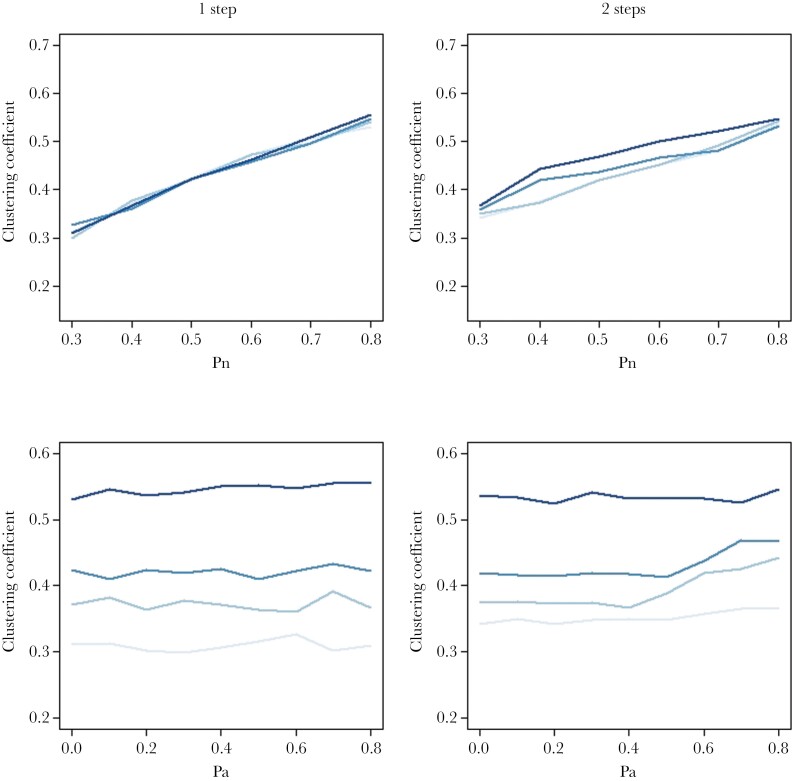
Clustering coefficient as a function of two social inheritance models and their parameters. Clustering coefficient was estimated at the last time step of each simulation and averaged across 100 replicates for the one-step model (or “vertical inheritance,” left panels) and the two-step model (or “vertical + horizontal inheritance,” right panels). For the two top panels, the four curves (from light to dark blue) correspond to different intensities for the social inheritance of avoidances (Pa = 0, 0.3, 0.6, 0.8). For the two bottom panels, the 4 curves (from light to dark blue) correspond to different intensities for the social inheritance of associates (Pn = 0.3, 0.4, 0.5, 0.8). For all panels, the probability to form random new ties (Pr) was 0.01, network size was fixed to 50 individuals, and simulations were initiated with random networks and run for 1000 time steps.

**Figure 4:**
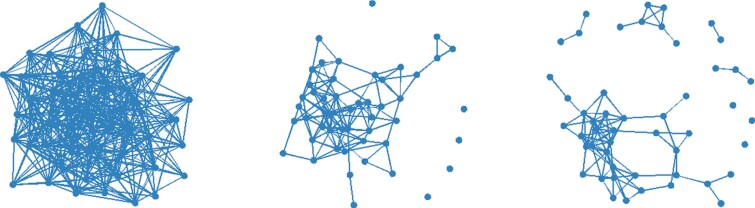
Examples of social networks (50 individuals) obtained in the two-step model under zero (Pa = 0, left), moderate (Pa = 0.4, middle), and high (Pa = 0.8, right) inheritance of avoidances. In these simulated examples, inheritance of associates is moderate (Pn = 0.4) and random connections are made with a probability Pr of 0.01.

In this model, Pn had a positive effect on the number of associates and mostly no effect on the number of avoidances. Pa had a positive effect on the number of avoidances and mostly no effect on the number of associates (Supplementary Figures 1 and 2). Regarding the network’s structure, Pn decreased modularity, and increased the clustering coefficient (Figures 2–4). In contrast, Pa had generally no impact on these metrics (Figures 2–4), except under high Pr values, where it had weak positive effects on modularity (Supplementary Figure 4). Pr generally increased the number of associates and avoidances and had negative impacts on the three metrics describing the associates’ network structure (Supplementary Figures 1–5).

### Vertical and horizontal transmission of social relationships (two-step model)

Networks generated under the two-step model had on average more affiliates and avoidance relationships, lower modularity and similar clustering coefficients compared to the one-step model (Figures 2–4). The impacts of Pa and Pn on most metrics were amplified, and some patterns only visible in the two-step models. In this model, Pn decreased the number of associates and had a nonlinear effect on the number of avoidances when Pa was strong (Pa = 0.6, 0.8), which peaked at intermediate values for Pn. In contrast to the one-step model, Pa decreased the number of associates when Pn had intermediate to high values, and strongly increased the number of avoidances. Regarding the associates’ network structure, the effects of Pn were qualitatively consistent with those found in the one-step model (negative for modularity, positive for clustering coefficient), but there were interactive effects with Pa (Figures 2–4). Indeed, the impacts of Pa on network metrics were particularly pronounced at intermediate values of Pn (Pn = 0.4–0.5). This is likely because Pn has a large impact on network density and that low and high values of Pn make networks that are very sparse/dense, thereby reducing the “degree of freedom” for Pa to influence it. We note that the impact of Pa on clustering coefficient was generally weak compared to Pn but increased in magnitude for lower values of Pr. Finally, Pr increased the number of avoidances and had weak negative effects on all three metrics describing the associates’ network structure but had no impact on the number of associates.

## DISCUSSION

While social avoidances may hide in plain sight, they are nonetheless a key driving force of social network structure in animals. Together, our series of social inheritance simulations provide a theoretical context within which social avoidance can be observed shaping the structure of animal social networks. Our results, along with previous theoretical and empirical studies, suggest that researchers of social evolution should incorporate avoidance into their thinking, and empiricists should make efforts to document and measure the extent of social avoidance.

Our simulations demonstrate that social avoidances can shape a social network’s structure when inherited from individuals’ entire social environments but not when only inherited from their mother. This absence of impact of maternally inherited avoidances on social structure is in contrast with the previous findings that maternal inheritance of affiliates increased density and clustering (Ilany and Akçay 2016) and can be explained by the fact that avoidances are an invisible form of negative relationships (Cartwright and Harary 1956; Ilany et al. 2013). For instance, when only considering the network of associations, a triad formed by a newborn, its mother and a mother’s avoidance will remain “open” regardless of whether the newborn inherits (−1) or not (0) its mothers’ avoidance. Inheriting avoidances therefore has very little impact at a local scale (triad), which is also why the clustering coefficient (related to the number of closed triangles in a network), is unaffected by Pa contrary to Pn. In contrast, the impact of social avoidances via two-step inheritance is due to the fact that newborns inherit avoidances from several non-independent individuals, all being the newborn’s mother’s associates. This is further supported by additional simulations in which maternal inheritance was zero and newborns only inherited avoidances from their randomly drawn network of associates. In these simulations, Pa had no impact on the various metrics of structure (Supplementary Figure 5). In nature, individuals likely do not form relationships with different individuals at random but will join an existing group or be introduced to a group via one of its members, here, assumed to be the mother. The reason why this process is important for avoidances to impact social structure is explained below.

Social inheritance of positive and negative relationships is a mechanism that intrinsically generates structural balance (the friend of my friend is my friend, and the enemy of my friend is my enemy), a force that is predicted to stabilize social structures (Ilany et al. 2013). Structural balance theory posits that when both positive and negative relationships occur, certain triads (+++, +--) are more stable than others (++- and ---). In our simulations, maternal inheritance strictly enforces structural balance, generating only +++ and +-- triads because individuals are simply “cloning” their mother’s network with a certain degree of relationship loss. In other words, maternal inheritance of avoidances only allows maintaining social structure at a local level (the triad mother, offspring, third individual). In contrast, inheriting avoidances from several associates (which already form +++ triads with the newborn and mother) not only occurs at a larger scale but imposes some compromises. Indeed, such inheritance pathway will statistically favor +-- over ++- triads but not strictly so, generating low levels of instability, which can later cause networks to split into substructures. Hence, because of these social constraints and instability, large-scale patterns emerge and the resulting networks are more complex and modular.

The social inheritance model clearly demonstrates how a simple set of rules can dictate who individuals associate with or avoid, and the direct consequences on the social network structure of a population. In doing so, we uncovered that social inheritance of both associations and avoidances can drive the evolution of structure in animal social networks (Ilany and Akçay 2016). We believe, therefore, that social avoidances should be considered as integral to the study of social evolution (Strickland et al. 2017, 2018; Piza-Roca et al. 2018; Strickland and Frere 2019). This is especially important given that properties of social networks (including avoidances) inform key aspects of population dynamics such as gene-culture coevolution (Whitehead et al. 2019), disease transmission (Hamede et al. 2009; Silk et al. 2019), emergence of cooperation (Aplin et al. 2014), and survival (Lehmann et al. 2016). We know, for instance, that network modularity can promote the evolution of cooperation (Marcoux and Lusseau 2013), yet the contribution of avoidances to this process remains unknown. It is possible that unraveling their contribution to population dynamic processes such as these will most likely result in fruitful discoveries.

We therefore make a call for more empirical work in this space. In study systems that utilize GPS data, this is relatively straightforward using measurements of spatial proximity and individual home ranges or other territory-based interaction data (Spiegel et al 2016; Strickland et al 2017). However, we could also extend this work to other frameworks. For example, spatial proximity tags might not give absolute spatial data but rather datapoints of close interactions between individuals. Thus, “avoidances” would not be calculated based on spatial absence, but rather on structural balance theory, whereby the likelihood of interactions could be calculated between all individuals, and thus rates of interactions and absences could be predicted. This would provide a baseline as to whether low levels (or complete absences) of interactions between two individuals might be within expected random chance based on the overall social network of the group, or, if they would be expected to interact more with one another based on their respective positions within the group. By utilizing social network theories, therefore, avoidances—and their inheritances—could be included in the study of populations for which only spatial proximity datapoints or group membership information is known.

Species may differ with respect to how they are structured and studied, but they may also differ in terms of their inheritance pathways. These will, of course, be based on their biology, including reproductive strategy and type of parental care they employ. Since avoidances are quite likely to be inherited culturally rather than (or in addition to) genetically, a range of inheritance pathways could be considered. For example, highly familial species might inherit avoidances from non-parental kin, while species engaging in alloparenting might inherit avoidances from non-relatives that have a high level of influence during development. Our own model allows for the inheritance pathway to be altered according to these factors, and we encourage their consideration in future studies on this topic.

Finally, the implications of avoidances and their inheritance likely extend beyond “global” social network traits. While our aim was to test the hypothesis of whether clustering and modularity might be influenced by social avoidances as well as social associations, further research could also focus on direct or individual traits. For example, the degree, strength or centrality of an individual within a social network might also be affected by their avoidances as well as the inherited avoidances of others. These, as well as global network traits, would likely have fitness consequences (Snyder-Mackler et al 2020), and thus should be included as part of investigations into the potential effects of avoidances on individuals as well as populations.

After decades of research on animal social networks, we still do not know the relative importance of associations versus avoidances in shaping social networks in different systems and/or taxa. This is despite the fact that both likely concurrently drive the diversity of social systems we see across the animal kingdom. Furthermore, our models support the argument that avoidances are not simply the default inverse of associations. In fact, they potentially come from different cognitive and/or selective processes and as such require different lines of research. For example, common or shared selective pressures favoring groups likely strengthen network associations (e.g., high-risk environments promoting associations in fish (Hasenjager and Dugatkin 2017)). However, risks associated with close contact to conspecifics likely drive avoidances (e.g., disease [Albery et al. 2020]). Across animal taxa, how common social avoidances are, their social inheritance, and their propensity to be shared among friends or close associates are all currently unknown. Similarly, we presently do not know whether individuals vary in their propensity to share relationships, whether this propensity is repeatable or plastic within individuals, nor the evolutionary significance of these patterns. Data repositories of social networks (Sah et al. 2019) hold great potential to begin unraveling how associations and social avoidances have worked hand in hand to shape the diversity of social systems we witness across the animal kingdom. To conclude, we hope this study will spark a shift in our approach to studying social evolution in animals, and in doing so discover the biological importance of the dark matter of sociality.

## Supplementary Material

arad088_suppl_Supplementary_Tables_1

arad088_suppl_Supplementary_Figures_1-2

## Data Availability

Analyses reported in this article can be reproduced using the scripts provided by Frère et al. (2023).
