# Supplementary material for: Social inheritance of avoidances shapes the structure of animal social networks
Source: Behav Ecol. 2023 Oct 23;35(1):arad088. doi: 10.1093/beheco/arad088 (PMC10773302; doi:10.1093/beheco/arad088)
Supplement: arad088_suppl_Supplementary_Tables_1 [file arad088_suppl_supplementary_tables_1.docx]

Supplementary Table 1. List of studies which utilized the Socprog program (Whitehead 2009b) to test for social structure and identified evidence for social avoidances among conspecifics.

| Publication Year | Author | Title | Taxon | Publication Title | Pages | Volume |
| --- | --- | --- | --- | --- | --- | --- |
| 2013 | Carter, K. D.; Seddon, J. M.; Frère, C. H.; Carter, J. K.; Goldizen, A. W. | Fission–fusion dynamics in wild giraffes may be driven by kinship, spatial overlap and individual social preferences | Angolan giraffe (Giraffa camelopardalis angolensis (now Giraffa giraffa angolensis)) | Animal Behaviour | 385-394 | 85 |
| 2010 | Leu, S. T.; Bashford, J.; Kappeler, P. M.; Bull, C. M. | Association networks reveal social organization in the sleepy lizard | Australian sleepy lizard (Tiliqua rugosa) | Animal Behaviour | 217-225 | 79 |
| 2011 | Parra, G. J.; Corkeron, P. J.; Arnold, P. | Grouping and fission–fusion dynamics in Australian snubfin and Indo-Pacific humpback dolphins | Australian snubfin dolphin (Orcaella heinsohni) and Indo-Pacific humpback dolphin (Sousa chinensis) | Animal Behaviour | 1423-1433 | 82 |
| 2012 | Mourier, J.; Vercelloni, J.; Planes, S. | Evidence of social communities in a spatially structured network of a free-ranging shark species | Blacktip reef shark (Carcharhinus melanopterus) | Animal Behaviour | 389-401 | 83 |
| 2010 | Frère, C. H.; Krützen, M.; Mann, J.; Watson-Capps, J. J.; Tsai, Y. J.; Patterson, E. M.; Connor, R.; Bejder, L.; Sherwin, W. B. | Home range overlap, matrilineal and biparental kinship drive female associations in bottlenose dolphins | Bottlenose dolphin (Tursiops aduncus) | Animal Behaviour | 481-486 | 80 |
| 2018 | Galezo, A. A.; Krzyszczyk, E.; Mann, J. | Sexual segregation in Indo-Pacific bottlenose dolphins is driven by female avoidance of males | Bottlenose dolphin (Tursiops aduncus) | Behavioral Ecology | 377-386 | 29 |
| 2010 | Jacoby, D. M. P.; Busawon, D. S.; Sims, D. W. | Sex and social networking: the influence of male presence on social structure of female shark groups | Catshark (Scyliorhinus canicula) | Behavioral Ecology | 808-818 | 21 |
| 2009 | Henzi, S.P.; Lusseau, D.; Weingrill, T.; Schaik, C.P.; Barrett, L. | Cyclicity in the structure of female baboon social networks | Chacma baboon (Papio hamadryas ursinus) | Behavioral Ecology and Sociobiology | 1015–1021 | 63 |
| 2017 | Surbeck, M.; Girard-Buttoz, C.; Boesch, C.; Crockford, C.; Fruth, B.; Hohmann, G.; Kevin, E.Langergraber; Zuberbühler, K.; Roman, M.Wittig; Mundry, R. | Sex-specific association patterns in bonobos and chimpanzees reflect species differences in cooperation | Chimpanzee (Pan troglodytes) and Bonobo (Pan paniscus) | Royal Society Open Science | e161081 | 4 |
| 2014 | Finger, A.; Patison, K. P.; Heath, B. M.; Swain, D. L. | Changes in the group associations of free-ranging beef cows at calving | Cow (Bos taurus) | Animal Production Science | 270-276 | 54 |
| 2016 | Stephenson, M. B.; Bailey, D. W.; Jensen, D. | Association patterns of visually-observed cattle on Montana, USA foothill rangelands | Cow (Bos taurus) | Applied Animal Behaviour Science | 42186 | 178 |
| 2013 | Abeyesinghe, S. M.; Drewe, J. A.; Asher, L.; Wathes, C. M.; Collins, L. M. | Do hens have friends? | Domestic chicken Gallus gallus domesticus | Applied Animal Behaviour Science | 61-66 | 143 |
| 2017 | Pearson, H. C.; Markowitz, T. M.; Weir, J. S.; Würsig, B. | Dusky dolphin (Lagenorhynchus obscurus) social structure characterized by social fluidity and preferred companions | Dusky dolphin (Lagenorhynchus obscurus) | Marine Mammal Science | 251-276 | 33 |
| 2014 | Best, E. C.; Dwyer, R. G.; Seddon, J. M.; Goldizen, A. W. | Associations are more strongly correlated with space use than kinship in female eastern grey kangaroos | Eastern grey kangaroo (Macropus giganteus) | Animal Behaviour | 44835 | 89 |
| 2017 | Strickland, K.; Levengood, A.; Foroughirad, V.; Mann, J.; Krzyszczyk, E.; Frère, C. H. | A framework for the identification of long-term social avoidance in longitudinal datasets | Eastern water dragon (Intellegama lesueurii) and bottlenose dolphin (Tursiops aduncus) | Royal Society Open Science | 170641 | 4 |
| 2011 | Buijs, S.; Keeling, L. J.; Vangestel, C.; Baert, J.; Vangeyte, J.; Tuyttens, F. A. M. | Assessing attraction or avoidance between rabbits: comparison of distance-based methods to analyse spatial distribution | European rabbit (Oryctolagus cuniculus) | Animal Behaviour | 1235-1243 | 82 |
| 2011 | Marsh, M. K.; McLeod, S. R.; Hutchings, M. R.; White, P. C. L. | Use of proximity loggers and network analysis to quantify social interactions in free-ranging wild rabbit populations | European rabbit (Oryctolagus cuniculus) | Wildlife Research | 44896 | 38 |
| 2017 | Rose, P. E.; Croft, D. P. | Social bonds in a flock bird: Species differences and seasonality in social structure in captive flamingo flocks over a 12-month period | Flamingo (Phoenicopterus spp) | Applied Animal Behaviour Science | 87-97 | 193 |
| 2021 | Brereton, J. E.; Fryer, J.; Rose, P. E. | Understanding sociality and behavior change associated with a nesting event in a captive flock of great white pelicans | Great white pelican (Pelecanus onocrotalus) | Zoo Biology | 386-397 | 40 |
| 2018 | Rose, P. E.; Croft, D. P. | Quantifying the social structure of a large captive flock of greater flamingos (Phoenicopterus roseus): Potential implications for management in captivity | Greater flamingo (Phoenicopterus roseus) | Behavioural Processes | 66-74 | 150 |
| 2019 | Gelardi, V.; Fagot, J.; Barrat, A.; Claidière, N. | Detecting social (in)stability in primates from their temporal co-presence network | Guinea baboon (Papio papio) | Animal Behaviour | 239-254 | 157 |
| 2016 | Napper, C. J.; Hatchwell, B. J. | Social dynamics in nonbreeding flocks of a cooperatively breeding bird: causes and consequences of kin associations | Long-tailed tit (Aegithalos caudatus) | Animal Behaviour | 23-35 | 122 |
| 2012 | Rutz, C.; Burns, Z.T.; James, R.; Ismar, S.M.H.; Burt, J.; Otis, B.; Bowen, J.; Clair, J.J.H. | Automated mapping of social networks in wild birds | New Caledonian crow (Corvus moneduloides) | Current Biology | R669-R671 | 22 |
| 2013 | Ilany, A.; Barocas, A.; Koren, L.; Kam, M.; Geffen, E. | Structural balance in the social networks of a wild mammal | Rock hyrax (Procavia capensis) | Animal Behaviour | 1397-1405 | 85 |
| 2017 | Mejía-Salazar, M. F.; Goldizen, A. W.; Menz, C. S.; Dwyer, R. G.; Blomberg, S. P.; Waldner, C. L.; Cullingham, C. I.; Bollinger, T. K. | Mule deer spatial association patterns and potential implications for transmission of an epizootic disease | Rocky Mountain mule deer (Odocoileus hemionus hemionus) | PLOS ONE | e0175385 | 12 |
| 2015 | Mahaffy, S. D.; Baird, R. W.; McSweeney, D. J.; Webster, D. L.; Schorr, G. S. | High site fidelity, strong associations, and long-term bonds: Short-finned pilot whales off the island of Hawai‘i | Short-finned pilot whale (Globicephala macrorhynchus) | Marine Mammal Science | 1427-1451 | 31 |
| 2008 | Gero, S.; Engelhaupt, D.; Whitehead, H. | Heterogeneous social associations within a sperm whale, Physeter macrocephalus, unit reflect pairwise relatedness | Sperm whale (Physeter macrocephalus) | Behavioral Ecology and Sociobiology | 143-151 | 63 |
| 2009 | Ramos-Fernández, G.; Boyer, D.; Aureli, F.; Vick, L. G. | Association networks in spider monkeys (Ateles geoffroyi) | Spider monkey (Ateles geoffroyi) | Behavioral Ecology and Sociobiology | 999-1013 | 63 |
| 2016 | Smith-Aguilar, S. E.; Ramos-Fernández, G.; Getz, W. M. | Seasonal Changes in Socio-Spatial Structure in a Group of Free-Living Spider Monkeys (Ateles geoffroyi) | Spider monkey (Ateles geoffroyi) | PLOS ONE | e0157228 | 11 |
| 2016 | Armansin, N. C.; Lee, K. A.; Huveneers, C.; Harcourt, R. G. | Integrating social network analysis and fine-scale positioning to characterize the associations of a benthic shark | Spotted wobbegong shark (Orectolobus maculatus) | Animal Behaviour | 245-258 | 115 |
| 2014 | Podgórski, T.; Lusseau, D.; Scandura, M.; Sönnichsen, L.; Jędrzejewska, B. | Long-Lasting, Kin-Directed Female Interactions in a Spatially Structured Wild Boar Social Network | Wild boar (Sus scrofa) | PLOS ONE | e99875 | 9 |
