# Supplementary material for: Social inheritance of avoidances shapes the structure of animal social networks
Source: Behav Ecol. 2023 Oct 23;35(1):arad088. doi: 10.1093/beheco/arad088 (PMC10773302; doi:10.1093/beheco/arad088)
Supplement: arad088_suppl_Supplementary_Figures_1-2 [file arad088_suppl_supplementary_figures_1-2.docx]

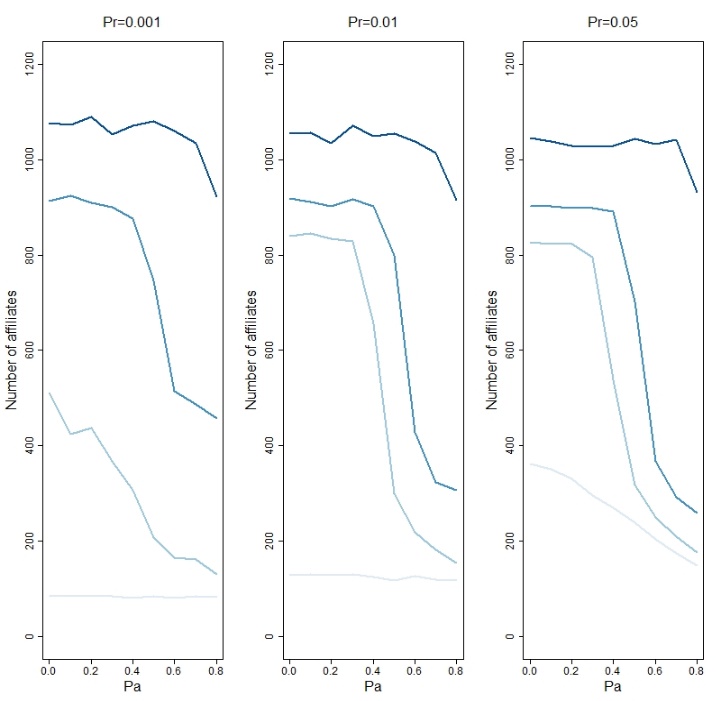

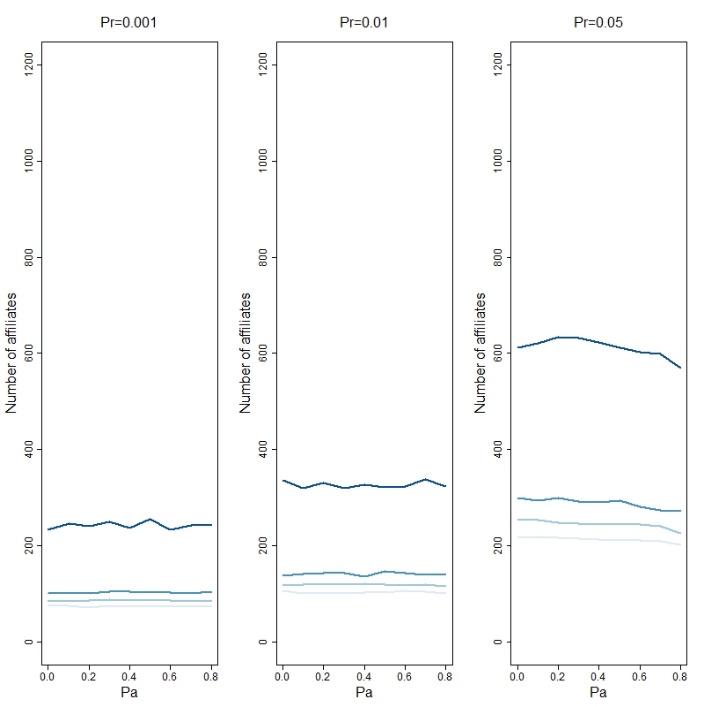

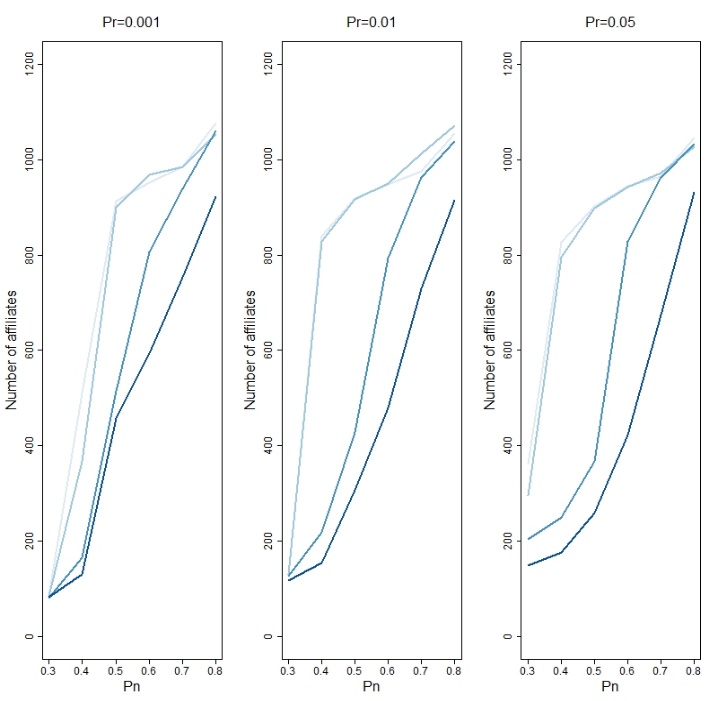

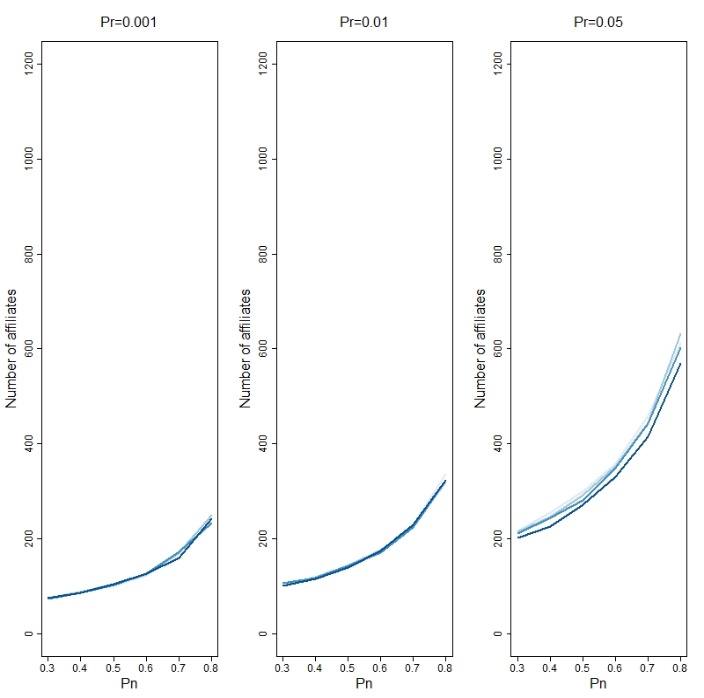


**Supplementary Figure 1.** Number of affiliates as a function of Pn (intensity of social inheritance of associates, top panels) and Pa (intensity of social inheritance of avoidances, bottom panels). Number of affiliates was estimated at the last time step of each simulation and averaged across 100 replicates for the 1-step model (or ‘vertical transmission”, left panels) and the 2-step model (or “vertical + horizontal transmission”, right panels). The 4 curves (from light to dark blue) correspond to different probabilities to form random new ties (Pr) For all panels, was 0.01, network size was fixed to 50 individuals, the simulations were initiated with random networks and run for 1000 time steps.


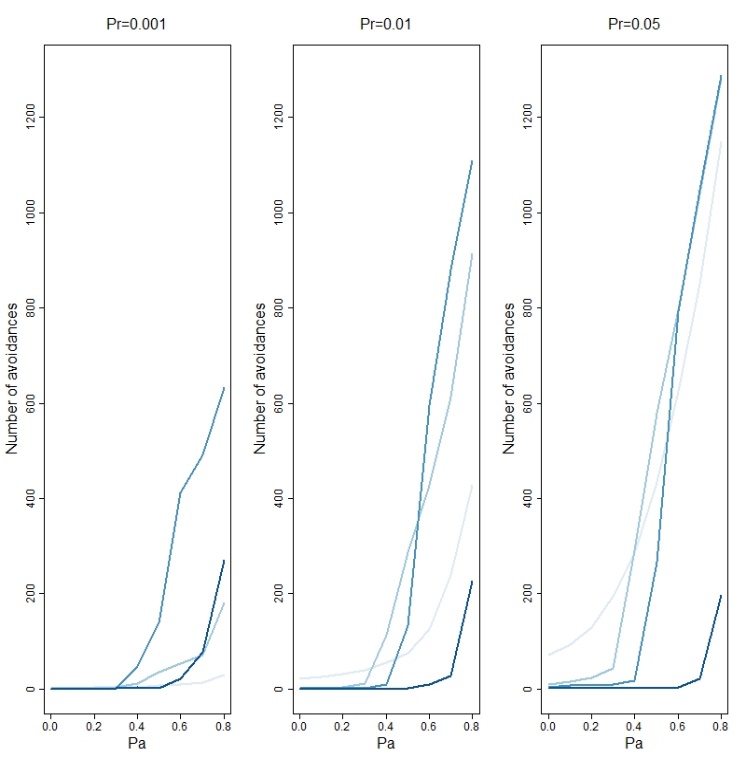

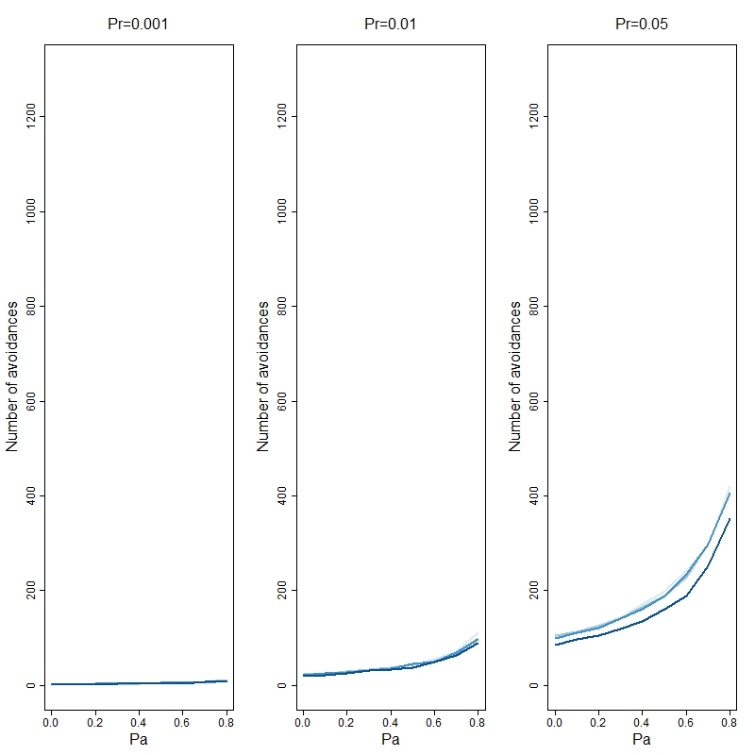

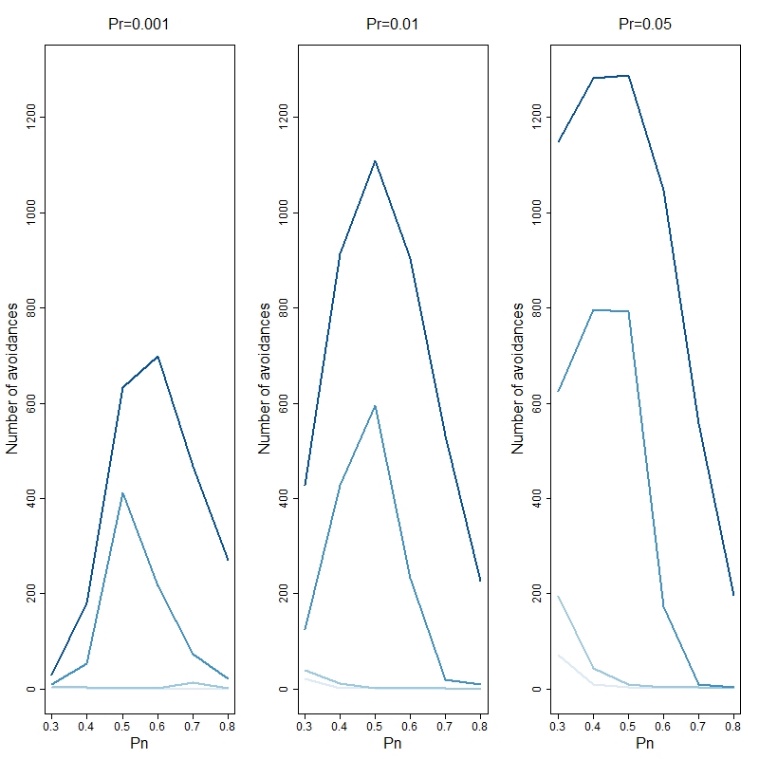

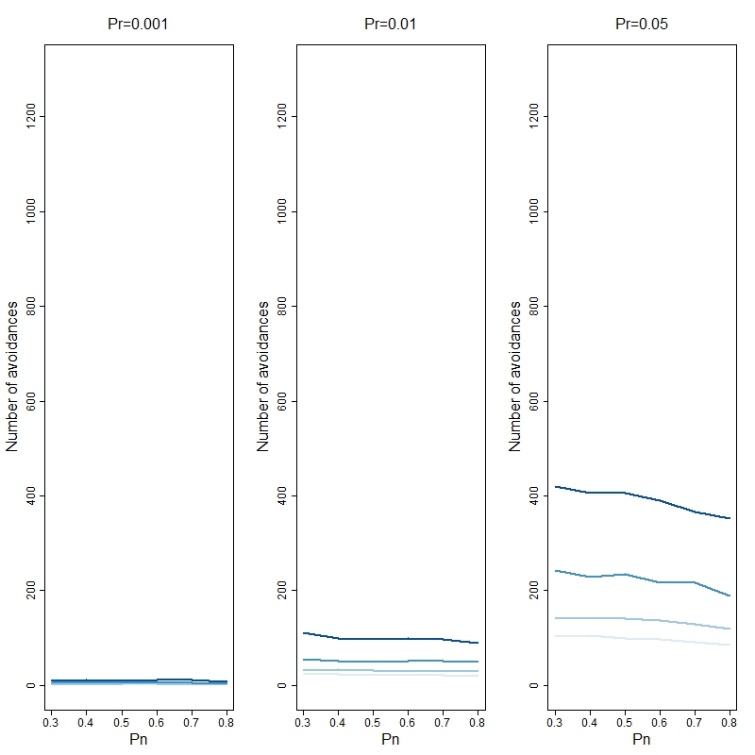
**Supplementary Figure 2.** Number of avoidances as a function of Pn (intensity of social inheritance of associates, top panels) and Pa (intensity of social inheritance of avoidances, bottom panels). Number of affiliates was estimated at the last time step of each simulation and averaged across 100 replicates for the 1-step model (or ‘vertical transmission”, left panels) and the 2-step model (or “vertical + horizontal transmission”, right panels). The 4 curves (from light to dark blue) correspond to different probabilities to form random new ties (Pr) For all panels, was 0.01, network size was fixed to 50 individuals, the simulations were initiated with random networks and run for 1000 time steps.

**Supplementary Figure 3.** Clustering coefficient as a function of Pn (intensity of social inheritance of associates, top panels) and Pa (intensity of social inheritance of avoidances, bottom panels). Number of affiliates was estimated at the last time step of each simulation and averaged across 100 replicates for the 1-step model (or ‘vertical transmission”, left panels) and the 2-step model (or “vertical + horizontal transmission”, right panels). The 4 curves (from light to dark blue) correspond to different probabilities to form random new ties (Pr) For all panels, was 0.01, network size was fixed to 50 individuals, the simulations were initiated with random networks and run for 1000 time steps.
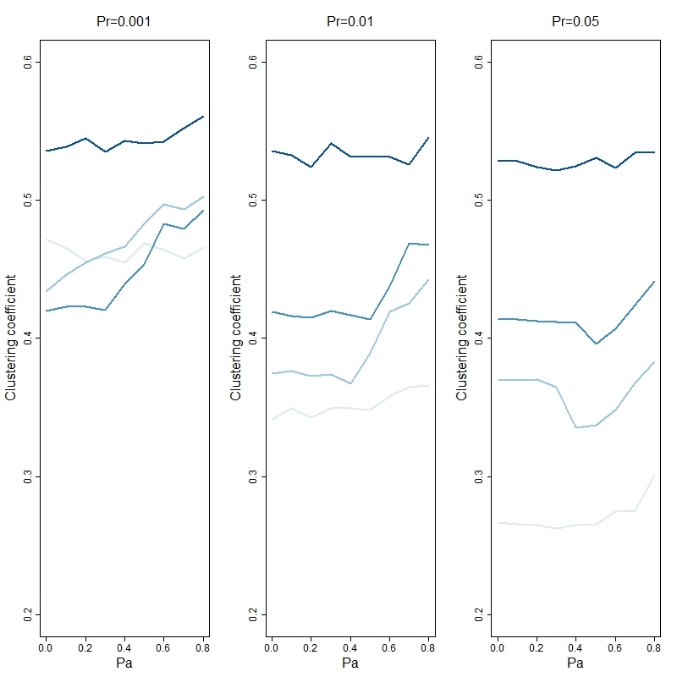

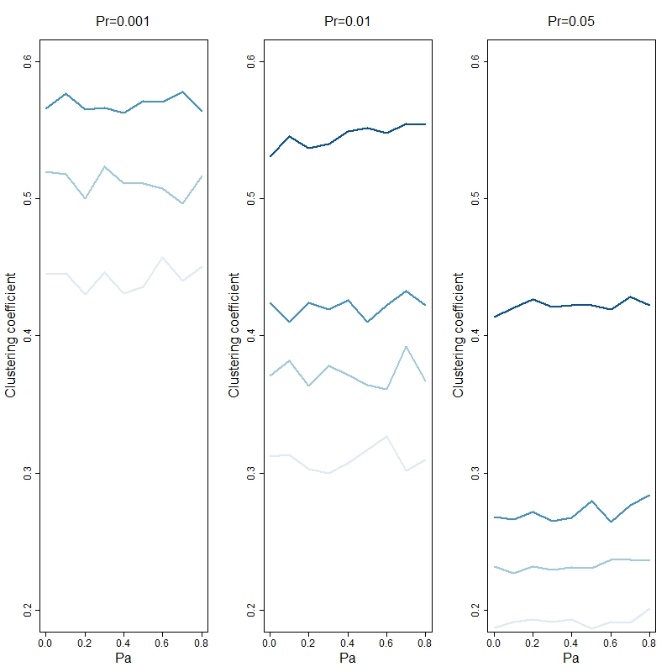

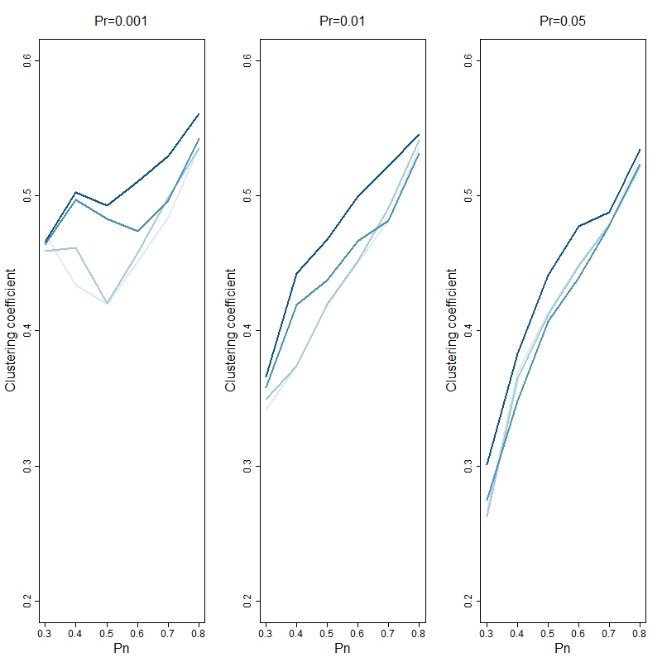

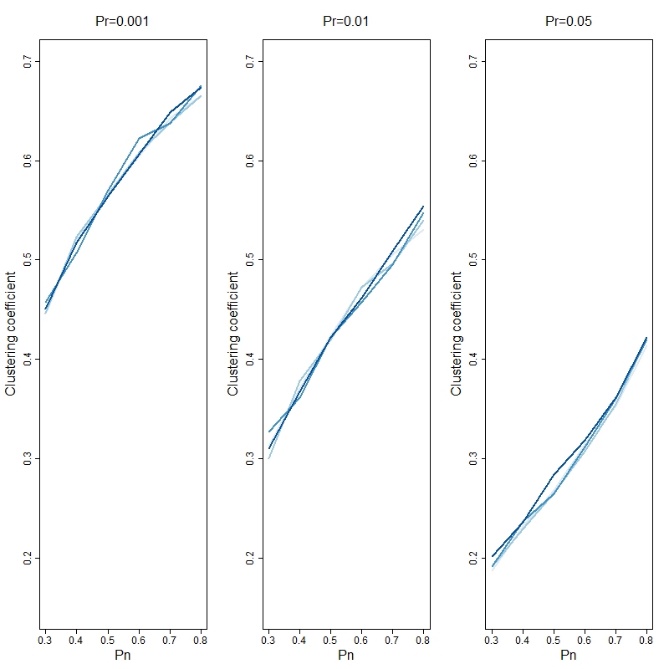


**Supplementary Figure 4.** Degree CV (coefficient of variation) as a function of Pn (intensity of social inheritance of associates, top panels) and Pa (intensity of social inheritance of avoidances, bottom panels). Number of affiliates was estimated at the last time step of each simulation and averaged across 100 replicates for the 1-step model (or ‘vertical transmission”, left panels) and the 2-step model (or “vertical + horizontal transmission”, right panels). The 4 curves (from light to dark blue) correspond to different probabilities to form random new ties (Pr) For all panels, was 0.01, network size was fixed to 50 individuals, the simulations were initiated with random networks and run for 1000 time steps.
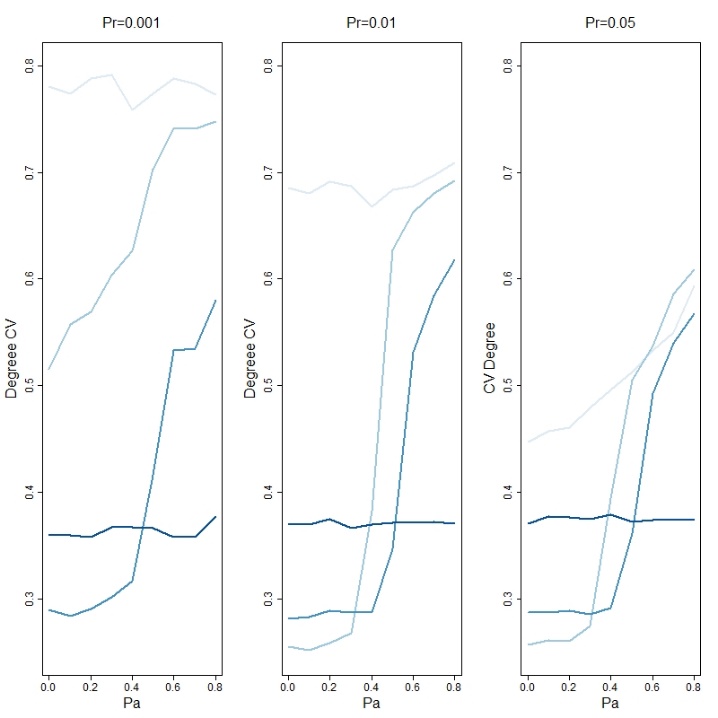

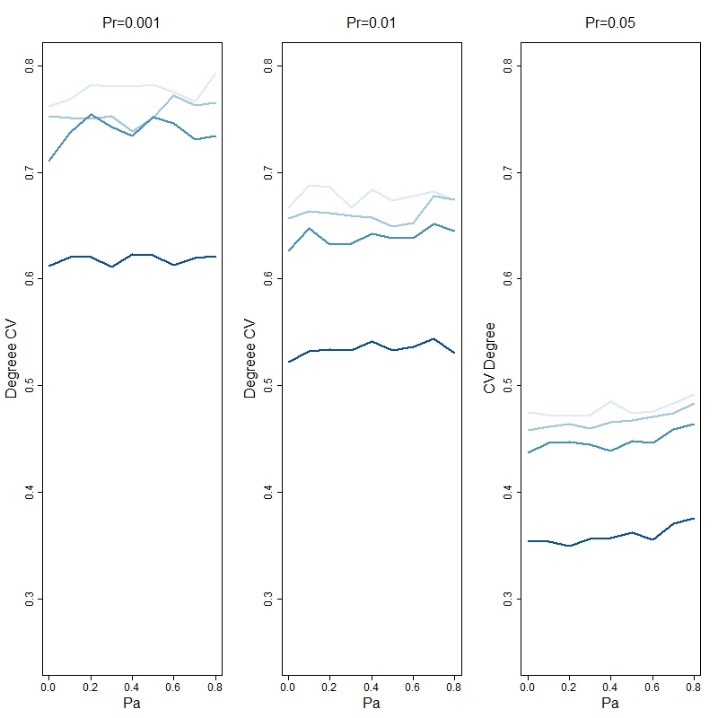

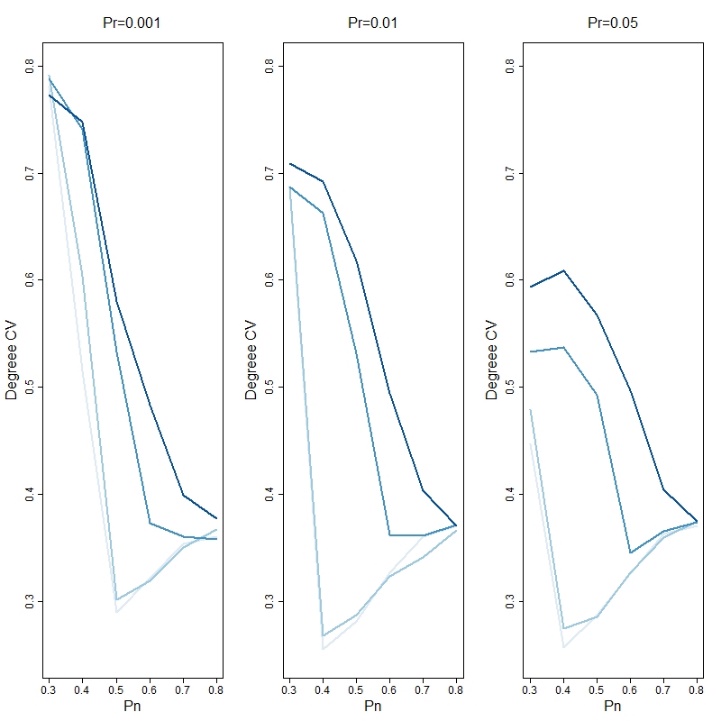

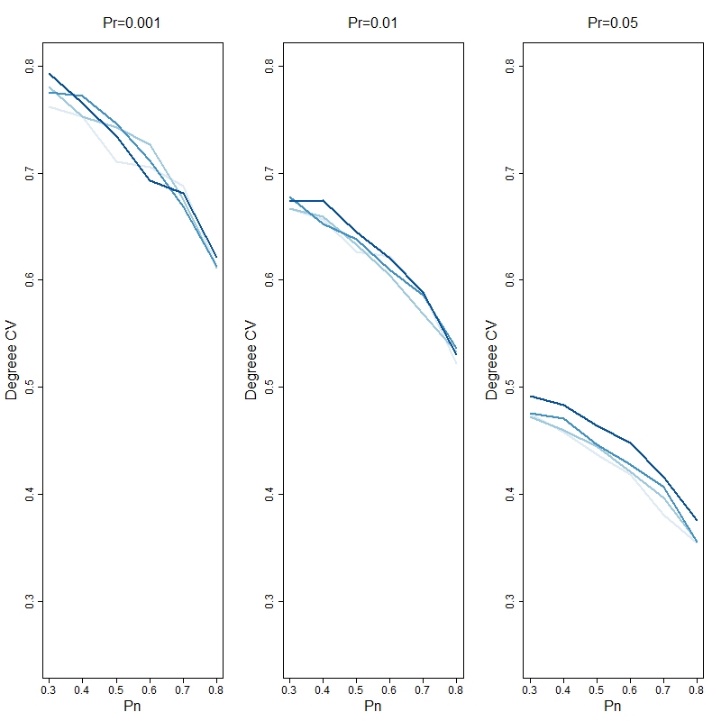


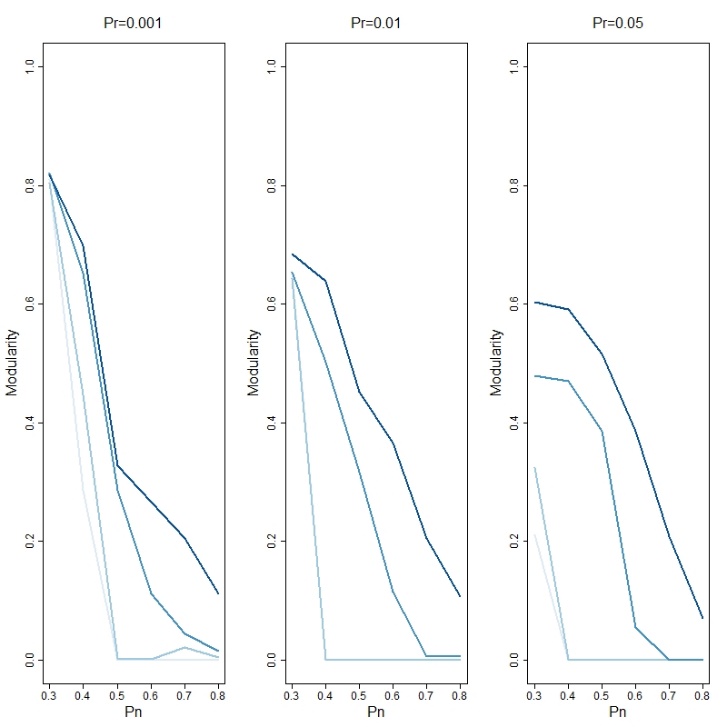

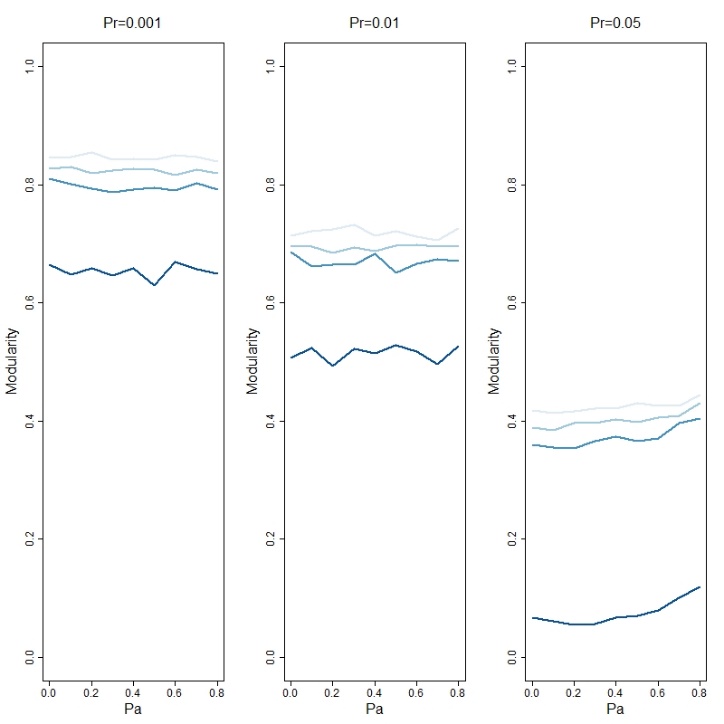

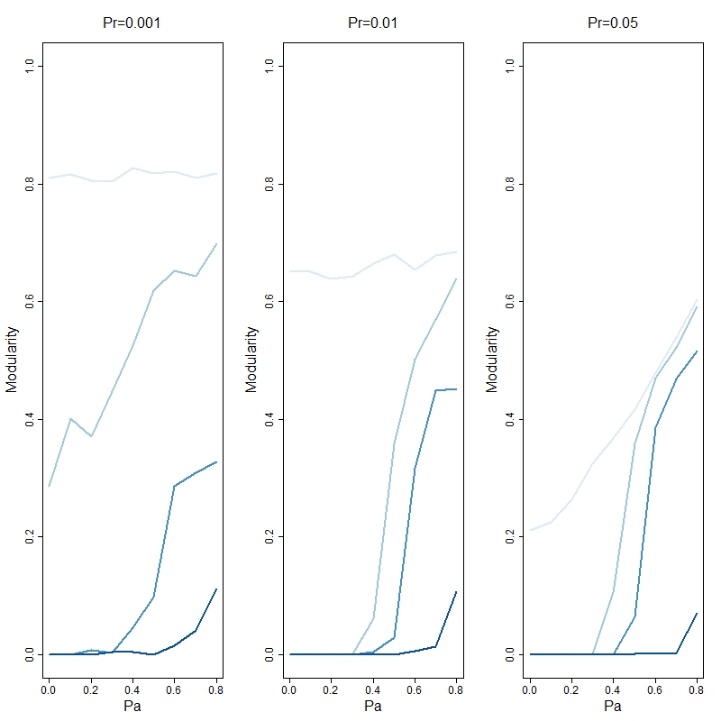

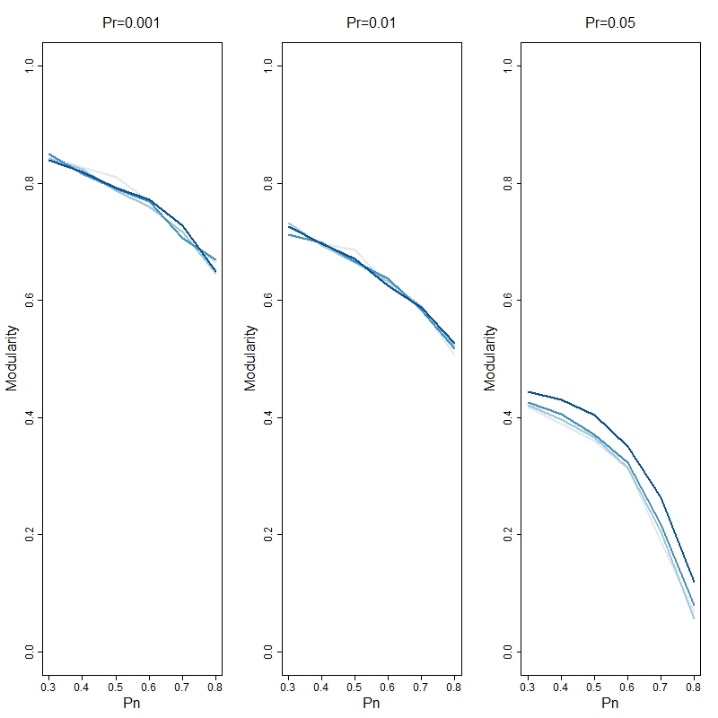


**Supplementary Figure 5.** Modularity as a function of Pn (intensity of social inheritance of associates, top panels) and Pa (intensity of social inheritance of avoidances, bottom panels). Number of affiliates was estimated at the last time step of each simulation and averaged across 100 replicates for the 1-step model (or ‘vertical transmission”, left panels) and the 2-step model (or “vertical + horizontal transmission”, right panels). The 4 curves (from light to dark blue) correspond to different probabilities to form random new ties (Pr) For all panels, was 0.01, network size was fixed to 50 individuals, the simulations were initiated with random networks and run for 1000 time steps.
